# Active Management Reduces the Incidence of Recurrent Pre-eclampsia and Improves Maternal and Fetal Outcomes in Women With Recurrent Pre-eclampsia

**DOI:** 10.3389/fmed.2021.658022

**Published:** 2021-04-29

**Authors:** Xin Dong, Min Han, Shahn Zeb, Mancy Tong, Xuelan Li, Qi Chen

**Affiliations:** ^1^Department of Obstetrics & Gynecology, First Affiliated Hospital of Xi'an Jiaotong University, Xi'an, China; ^2^School of Medicine, Xi'an Jiaotong University, Xi'an, China; ^3^Department of Obstetrics, Gynecology and Reproductive Sciences, Yale University, New Haven, CT, United States; ^4^Department of Obstetrics & Gynecology, The University of Auckland, Auckland, New Zealand

**Keywords:** pre-eclampisa, recurrence, neonatal outcomes, active management, dose aspirin

## Abstract

**Background:** Women with previous pre-eclampsia are at an increased risk of developing recurrent pre-eclampsia. Intervention with low dose aspirin had been recommended to reduce the incidence of recurrent pre-eclampsia. However, the association between interventions and maternal and neonatal outcomes in subsequent pregnancies in women with previous pre-eclampsia has not been fully studied.

**Methods:** In this prospective study, a total of 41 patients with previous pre-eclampsia received low dose aspirin and active management (including psychological and physiological intervention), between 10 to 28 weeks until 32 to 34 weeks in our regional referral hospital. The recurrence of pre-eclampsia, and maternal and neonatal outcomes in this pregnancy were analyzed and compared to our previous study which reported a 60% recurrence of pre-eclampsia in our regional referral hospital.

**Results:** Thirteen women with previous pre-eclampsia developed recurrent pre-eclampsia. The time of onset or severity of pre-eclampsia in the previous pregnancy was not associated with the incidence of recurrent pre-eclampsia. The time of onset of previous pre-eclampsia was also not associated with the time of onset in subsequent pre-eclampsia. However, the number of severe recurrent pre-eclampsia was significantly reduced, compared to their first pregnancies. The number of SGA and stillbirth/neonatal death was also significantly reduced in recurrent pre-eclampsia that was actively managed, compared to their first pregnancies.

**Conclusion:** Despite the small sample size included in this study, our study demonstrates that active obstetric management reduces the incidence of recurrent pre-eclampsia, compared to our previous study, and reduces the severity of recurrent pre-eclampsia. It also improves neonatal outcomes in recurrent pre-eclampsia. However, because of no controls in this study, our findings need to confirmed by a case-control or randomized clinical trial study.

## Introduction

Pre-eclampsia is a human-specific pregnancy disorder that is a leading cause of maternal and neonatal mortality and morbidity. It is clinically apparent only after 20 weeks of gestation or within the first 4–6 weeks postpartum and is responsible for about 70,000 maternal deaths and 500,000 fetal and neonatal deaths annually worldwide. Although the exact pathogenesis of pre-eclampsia is still unclear, a number of risk factors including maternal age, obesity and history of pre-eclampsia have been well-documented.

Although some studies have reported that pre-eclampsia is more common in the first pregnancy, the incidence of recurrent pre-eclampsia in a subsequent pregnancy is around 13 to 65% (regional or ethnicity dependent) (1–3). Our previous study also reported that the incidence of recurrent pre-eclampsia is 60% in our regional referral hospital, with a large number of severe and early onset cases (4). To date, no single biomarker has been clinically useful for the prediction of the recurrent pre-eclampsia. In addition, a history of pre-eclampsia also does not predict the recurrence of pre-eclampsia in subsequent pregnancy. Therefore, clinical intervention in pregnant women with a history of pre-eclampsia is recommended in order to reduce the chances of recurrence.

It has been reported that clinical intervention with low dose aspirin, low molecular weight heparin, calcium supplementation, or vitamin D supplementation has some benefits for reducing the incidence of recurrent pre-eclampsia (5–8). Studies reported that intervention with low dose aspirin or low molecular weight heparin can, respectively, reduce 30 and 20% of recurrent pre-eclampsia in women with previous pre-eclampsia (6–8). Another study reported that vitamin D supplementation can reduce 15% of recurrent pre-eclampsia (5). However, most of these studies only focused on investigating the role of these interventions in reducing the incidence of recurrent pre-eclampsia. Whether there is a difference in clinical characteristics between women with recurrent pre-eclampsia and without recurrent pre-eclampsia, and whether these interventions can affect maternal and neonatal outcomes such as the time of onset or severity of recurrent pre-eclampsia in women with previous pre-eclampsia have not been fully studied. The One Child Policy in China ended in October 2015 resulting in a significant increase in the number of subsequent pregnancies. Therefore, we undertook this prospective study to investigate whether low dose aspirin and active obstetric management can affect the incidence of recurrent pre-eclampsia in women with previous pre-eclampsia. In women who had recurrent pre-eclampsia, we also compared the difference in maternal and neonatal outcomes between first pregnancy and subsequent pregnancy in which low dose aspirin was administered and patients were actively managed.

## Materials and Methods

This investigation conforms to the principles outlined in the Declaration of Helsinki. This prospective study was approved by the Ethics Committee of First Hospital of Xi'an Jiaotong University, China (ref 2018-115).

### Study Population

This prospective study was performed at one of the university teaching hospitals serving a diverse urban and rural population of approximately 8 million people in China. The First Hospital of Xi'an Jiaotong University is the main maternal care referral hospital in Xi'an city and large numbers of women with pre-eclampsia, in particular those women with high risk such as previous pre-eclampsia are referred to this hospital. A total number of 50 pregnant women with previous pre-eclampsia who came or referred to our high-risk obstetrics clinic were enrolled in this study between 2015 and 2018. All the women were randomly recruited. Women with multiple pregnancies, and underlying medical disorders such as pulmonary and cardiac disease were excluded from this study. Pregnancies conceived by *in vitro* fertilization (IVF) were also excluded.

Of the 44 enrolled pregnant women, two patients were lost during the follow-up in the third trimester and one patient had a spontaneous abortion at 19 weeks of gestation due to cervical incompetence. The remaining 41 patients gave a birth at our hospital and data were collected and analyzed.

Data including maternal age, time of onset of previous and/or current pre-eclampsia, severity of previous and/or current pre-eclampsia, gestational week at delivery of previous and current pregnancies, neonatal results in previous pregnancy, interval between the two pregnancies, and pre-pregnancy BMI in this pregnancy were recorded by obstetricians.

Pre-eclampsia was defined as a maternal systolic blood pressure ≥140 mmHg and/or diastolic blood pressure ≥90 mmHg measured on two occasions separated by at least 6 h, and proteinuria >300 mg in a 24 h period, or impaired liver function and lower platelet count, after 20 weeks of gestation in accordance with guidelines of the International Society for the Study of Hypertension in Pregnancy (ISSHP) in 2018 (9). Maternal systolic blood pressure ≥160 mmHg and/or diastolic blood pressure ≥110 mmHg was defined as severe pre-eclampsia. Pre-eclampsia occurring earlier than 34 weeks of gestation was defined as early onset.

Small for gestational age (SGA) was defined birthweight under 10th percentile using the WHO fetal growth chart based on the gestational age (10).

### Active Management (Including Inpatient and Outpatient Management)

#### Psychological and Physiological Intervention

To reduce the fear and anxiety in this current pregnancy for patients who were recruited to this study, obstetricians in our high-risk clinical team shared the experiences of women with previous pre-eclampsia who did not develop pre-eclampsia in subsequent pregnancies. The experiences included the importance of antenatal visits or close monitoring including home blood pressure monitoring through the duration of pregnancy as well as obstetricians or midwives' contact. In addition, obstetricians also educated the family members, in particular the husband or partner about the risks of recurrent pre-eclampsia and encouraged family members to build a relaxed environment for pregnant women both at home and in the workplace. These pregnant women were asked to visit a prenatal clinic every 2–3 weeks, dependent on the maternal and fetal conditions until 34 weeks. After 34 weeks, they were asked to visit the prenatal clinic every week. Furthermore, because BMI is one of the main risk factors for pre-eclampsia, obstetricians recommended pregnant women who were overweight (BMI > 23 kg/m^2^) or obese (BMI > 27.50 kg/m^2^) at the time of first prenatal clinic to lose weight until their BMI neared 23 kg/m^2^ by monitoring their diet and exercise with guidance from a nutritionist.

#### Medical Intervention

A total of 41 women with previous pre-eclampsia received low-dose aspirin prophylaxis (100 mg/day) between 10 weeks and 28 weeks of gestation until 32 to 34 weeks. Of them, 8 patients also received Low Molecular Weight Heparin (LMWH) (4100IU/day) when they were diagnosed with APS (antiphospholipid antibodies (aPLs) positive). The duration of treatment was up to 34 weeks of gestation for those who did not develop recurrent pre-eclampsia or up to 37 weeks of gestation or 12 h before delivery for those who developed recurrent pre-eclampsia. Patients were also given antihypertensive drugs such as labetalol or nifedipine to control the blood pressure when their systolic or diastolic blood pressure was over 130 or 80 mmHg measured by 24 h ambulatory blood pressure monitoring, even they have not developed pre-eclampsia yet.

Prescribing antihypertensive drug to women with a history of pre-eclampsia, when their systolic or diastolic blood pressure is over 130 or 80 mmHg measured by 24 h ambulatory blood pressure monitoring, is our hospital protocol.

#### General Management

Based on the results from the routine liver and renal function tests and echocardiography (ECG) monitoring, 24 h ambulatory blood pressure measurement or liver and kidney ultrasound were recommended to individual patients if it was necessary. In addition, since not all the patients stayed in the hospital, daily home blood pressure measurements were recommended to all patients in order to monitor the trend of blood pressure to see whether blood pressure increased or the maternal conditions deteriorated. At least, one senior obstetrician was able to be contacted during the study period, if women did not stay in the hospital.

#### Statistical Analysis

Data were presented as mean and standard deviation (SD) or number or percentage as appropriate. The statistical differences in maternal age, gestational week at diagnosis, gestational week at delivery, and stillbirth/neonatal death between first pregnancy and subsequent pregnancy were assessed by the Mann-Whitney U-test or Fisher's exact test using the Prism software package. The comparison between the severity or time of onset between first pre-eclampsia and recurrent pre-eclampsia was assessed by McNemar test using OpenEpi (Open Source Epidemiologic Statistics for Public Health, Version. www.OpenEpi.com). *P* < 0.05 were considered significant.

## Results

Overall, of the 41 recruited patients in this study, 13 (32%) patients developed recurrent pre-eclampsia. The clinical parameters of patients who developed recurrent pre-eclampsia and those who did not are summarized in Table 1. The mean systolic or diastolic blood pressure was 153 ± 15 or 100 ± 8 mmHg, respectively, in patients who developed recurrent pre-eclampsia. There was no statistical difference in maternal age, BMI, and interval between the two pregnancies (*p* > 0.05). The number of patients who had BMI ≥23 kg/m^2^ in the no recurrence group was not statistically different to that in the recurrent group (38 vs. 25%, *p* = 0.468). The mean start-time of aspirin intervention was significantly earlier in patients who did not develop recurrent pre-eclampsia compared to patients who did (13 vs. 18 weeks, *p* = 0.01). The gestational weeks at delivery and birthweight were significantly earlier or lower in patients who developed recurrent pre-eclampsia than in patients who did not (*p* < 0.001), but the number of SGA infants was not statistically different between the two groups (*p* = 0.0951, Table 1). In addition, the number of infants who went to the neonatal intensive care unit (NICU) was significantly higher in patients in the recurrence group than the other (84 vs. 25%, *p* < 0.001).

**Table 1:** Clinical parameters of patients with and without recurrent pre-eclampsia.

	**Recurrent pre-eclampsia (*n* = 13)**	**No recurrent pre-eclampsia (*n* = 28)**	***P*-value**
Maternal age (year, mean ± SD)	32.3 ± 3.6	31.3 ± 3.6	0.531
Pre-pregnancy BMI (kg/m^2^) in 2nd pregnancy	22.81 ± 2.5	23.04 ± 2.9	0.541
Interval between two pregnancies (years, mean ± SD)	5.7 ± 3.8	4.2 ± 3.0	0.200
Systolic blood pressure (mmHg, mean ± SD)	153 ± 15	<140	<0.001
Diastolic blood pressure (mmHg, mean ± SD)	100 ± 8	<90	<0.001
Time of starting intervention (weeks/mean ± SD)	18 ± 7.7	13 ± 3.8	<0.001
**Gravida**			
*n* = 2	7 (54%)	14 (50%)	>0.999
*n* ≥ 3	6 (46%)	14 (50%)	
Delivery week (weeks, mean ± SD)	34^+2^ ± 2^+2^	38^+1^ ± 1^+1^	<0.001
Birthweight (g/mean ± SD)	2,177 ± 747	3,171 ± 453	<0.001
SGA (*n*, %)	2 (15%)	0 (0%)	0.0951
Admission to NICU (*n*, %)	11 (84%)	7 (25%)	<0.001

We then analyzed the associations between the severity or the time of onset of previous pre-eclampsia and the incidence of recurrent pre-eclampsia. There were 9 (47%) patients with previous severe pre-eclampsia (*n* = 19) who developed recurrent pre-eclampsia (Table 2). In addition, there were 10 (35%) patients with previous early onset pre-eclampsia (*n* = 28) who developed recurrent pre-eclampsia (Table 2). Thus, there was no association between the time of onset or severity of pre-eclampsia in first pre-eclampsia and the incidence of recurrent pre-eclampsia and the time of onset or severity of pre-eclampsia in first pre-eclampsia (*p* = 0.492 or *p* = 0.091, respectively, Table 2).

**Table 2:** The association between severity or the time of onset in previous pre-eclampsia and developing recurrent pre-eclampsia.

**Previous pre-eclampsia (*n* = 41)**	**Recurrent pre-eclampsia (*n*, %)**	**No recurrent pre-eclampsia (*n*, %)**	***p*-value**
Severe form (*n* = 19)	9 (47%)	10 (53%)	0.091
Mild form (*n* = 22)	4 (18%)	18 (82%)	
Early onset (*n* = 28)	10 (35%)	18 (65%)	0.492
Late onset (*n* = 13)	3 (23%)	10 (77%)	

In patients with recurrent pre-eclampsia (*n* = 13), 9 (70%) patients developed early onset pre-eclampsia in the subsequent pregnancy and 3 (23%) patients developed severe pre-eclampsia. We next compared the time of onset or severity of pre-eclampsia in the first pregnancy with that of the subsequent pregnancy in these patients (Table 3). Six (66%) patients with early onset pre-eclampsia in their first pregnancy developed early onset pre-eclampsia in subsequent pregnancy. Four (100%) patients with late onset pre-eclampsia in the first pregnancy developed early onset pre-eclampsia in their subsequent pregnancy. There was no statistical difference in the incidence of early onset recurrent pre-eclampsia in patients with early onset previous pre-eclampsia (*p* = 0.363, Table 3). In addition, 3 (23%) patients with severe pre-eclampsia and 1 (25%) patients with mild pre-eclampsia in the first pregnancy developed severe pre-eclampsia in subsequent pregnancy. The incidence of developing severe recurrent pre-eclampsia was significantly lower (*p* = 0.035, Table 3) in patients with previous pre-eclampsia who were received active management. There was no statistical difference in gestational weeks at delivery between first and subsequent pregnancies in patients with recurrent pre-eclampsia (*p* = 0.807). A total of 4 (30%) stillbirth/neonatal death were recorded in their first pregnancy in patients with recurrent pre-eclampsia, which was significantly higher than the number of stillbirth/ neonatal death in subsequent pregnancy (0%) (*p* < 0.0001). There was also no difference in birthweight between the first and subsequent pregnancies in patients with recurrent pre-eclampsia (2,224 vs. 2,177 g, *p* = 0.275).

**Table 3:** Severity or time of onset between first and subsequent pregnancy in patients with recurrent pre-eclampsia.

		**Subsequent pregnancy (*****n*** **=** **13)**		
		**Severe form (*n*, %)**	**Mild form (*n*, %)**	***P*-value**
First pregnancy (*n* = 13)	Severe form (*n* = 9)	3 (27%)	6 (73%)	0.035
	Mild form (*n* = 4)	1 (28%)	3 (72%)	
		**Early onset (*****n*****, %)**	**Late onset (*****n*****, %)**	
First pregnancy (*n* = 13)	Early onset (*n* = 9)	6 (75%)	3 (25%)	0.363
	Late onset (*n* = 4)	4 (100%)	0 (0%)	

We also analyzed the postnatal outcomes. There was no stillbirth or neonatal death, and the mean birthweight was 2,775 g. The mean gestational age at delivery was 36^+4^. However there were 18 infants who were admitted to NICU. Of 41 pregnant women with a history of pre-eclampsia, there were 25 (61%) women who received antihypertensive drugs. Of those that received the antihypertensive drugs, 10 women developed recurrent pre-eclampsia including 3 severe form and 7 mild form. The mean gestational age at delivery for these 3 women was 36^+3^, and the mean birthweight was 2,775 g. All these infants who were born from 3 women with severe pre-eclampsia were admitted to NICU. In contrast, in 16 women with a history of pre-eclampsia who did not receive antihypertensive drugs, 3 women developed recurrent pre-eclampsia, including 1 severe and 2 mild form.

## Discussion

In this prospective and observational study, we report that the incidence of recurrent pre-eclampsia was 32% in patients with a history of pre-eclampsia who received active management including low dose aspirin. We also found that severity or the time of onset of pre-eclampsia in the first pregnancy was not associated with the development of recurrent pre-eclampsia. In addition, early onset pre-eclampsia in the first pregnancy was not associated with early onset pre-eclampsia in the recurrent pre-eclamptic group. However, active management including the low dose of aspirin can reduce the rate of severe recurrent pre-eclampsia, compared to their first pregnancies.

Although a history of previous pre-eclampsia does not predict the recurrence of pre-eclampsia in subsequent pregnancies, previous pre-eclampsia is one of the risk factors for the development of recurrent pre-eclampsia. Clinical intervention including low dose aspirin has been recommended by The American College of Obstetricians and Gynecologists (ACOG) and other guidelines to use in pregnant women with previous pre-eclampsia in order to reduce the incidence of recurrent pre-eclampsia (11, 12). Aspirin, a cyclooxygenase inhibitor with anti-inflammatory and antiplatelet properties has been reported to prevent or delay the onset of pre-eclampsia as well as reduce stillbirth and preterm birth [reviewed in ACOG Committee Opinion No. 743 (11)]. The incidence of recurrent pre-eclampsia is around 13% to 65% (regional or ethnicity dependent) in subsequent pregnancies (1–3). We previously reported that 60% of women experienced recurrent pre-eclampsia in our hospital when they did not receive the current active management protocol (4). The higher incidence of recurrent pre-eclampsia is because that our hospital is a referral hospital and a large number of severe or early onset pre-eclampsia are referred to our hospital. We also followed the international protocol in the previous study (4). However, in our current study, we found the incidence of recurrent pre-eclampsia was 32%, when patients with previous pre-eclampsia received active management including low dose aspirin and antihypertensive drugs when their systolic or diastolic blood pressure was over 130 or 80 mmHg. Our data showed a 53% reduction in developing recurrent pre-eclampsia compared to our previous study (4). Our finding is better than a recent study that reported a 30% reduction in recurrent pre-eclampsia when low dose aspirin was given (6).

Pre-eclampsia is divided into severe and mild forms, or early onset and late onset forms according to the severity or the time of onset. It has previously been reported that women with early onset or severe pre-eclampsia in their first pregnancy are at an increased risk of developing recurrent pre-eclampsia (13, 14). However, our previous study reported that the time of onset or severity of pre-eclampsia in their first pregnancy was not associated with the incidence of recurrent pre-eclampsia (4) suggesting the time of onset or severity of pre-eclampsia are not risk factors for recurrent pre-eclampsia (6). In our current study, we also found that the severity or the time of onset in previous pre-eclampsia was not associated with developing recurrent pre-eclampsia in patients who received active management. A future study with a large sample size should be performed to confirm our findings. In addition, although our previous study (4) suggested that women with early onset pre-eclampsia in their first pregnancy are more likely to experience early onset recurrent pre-eclampsia, in our current study we found that the time of onset in previous pre-eclampsia was not associated with the time of onset of recurrent pre-eclampsia in patients who received active management. However, we did find that the number of severe recurrent pre-eclampsia cases was significantly reduced in patients who received active management, compared to their first pregnancy. This data suggests that active management including low dose aspirin can reduce the severity of recurrent pre-eclampsia.

It is well-reported that the time of intervention is very important. ACOG recommends that the optimal intervention time is between 12 and 28 weeks of gestation (11). A recent meta-analysis reported that when low-dose aspirin was given after 16 weeks, there was only a modest reduction in pre-eclampsia in women with high risks of pre-eclampsia (15). Another meta-analysis found a reduction in pre-term pre-eclampsia when low-dose aspirin was given before 16 weeks (16). In contrast, another meta-analysis study recently found that there was no difference in the benefits of low-dose aspirin on the prevention of pre-eclampsia regardless of whether the treatment started before or after 16 weeks of gestation (17). For women with previous pre-eclampsia, starting from 12 weeks of gestation for aspirin intervention was recommended by USPSTE study (the US Preventive Services Task Force) (8). In our current study, the majority of patients (75%) received the intervention at 10–12 weeks of gestation. Due to the limited sample size we were not able to analyse the difference in recurrent pre-eclampsia between patients who received the intervention at 10–12 weeks and after 12 weeks. However, our data showed that the intervention time in patients without recurrent pre-eclampsia was significantly earlier than that in patients with recurrent pre-eclampsia (13 vs. 18 weeks). This suggests that earlier intervention may have benefits in reducing the incidence of recurrent pre-eclampsia.

Previous studies have reported that even in the absence of recurrent pre-eclampsia, women with a history of pre-eclampsia are at a higher risk of delivering an SGA infant in their subsequent pregnancy (18). That study showed a 48% increase in delivery of an SGA infant in the subsequent pregnancy in patients who did not develop recurrent pre-eclampsia. However, in our current study we found that no patient in the no recurrence group delivered an SGA infant in their subsequent pregnancy. In addition, only 2 (15%) patients with recurrent pre-eclampsia delivered SGA infants in their subsequent pregnancy. This data suggests that even in patients with recurrent pre-eclampsia, active management including low-dose aspirin can significantly reduce the incidence of delivering SGA infants. In addition, we found that there were 4 stillbirth/neonatal deaths in patients with recurrent pre-eclampsia in their first pregnancy, but there was none stillbirth/neonatal death in patients with recurrent pre-eclampsia in their subsequent pregnancy when they received active management.

There are some limitations in this prospective study. First, the sample size was relatively small, as this study was performed in a single maternal care referral hospital. Our study should be repeated with a larger sample size to confirm our findings including maternal and fetal outcomes in the future with multi maternal centers. Second, due to the clinical practice protocol in our hospital, we did not have a control group that did not receive active management. Third, some of the clinical parameters such as BMI in the first pregnancy were not available. Whether controlling weight gain in subsequent pregnancy had benefits on reducing recurrent pre-eclampsia needs to be studied in the future.

## Conclusion

Despite the small sample size included in this study, in this prospective study, we demonstrate that active management including administration of low dose aspirin starting from 10 to 12 weeks can reduce the incidence of recurrent pre-eclampsia in women with previous pre-eclampsia, compared to our previous study which reported 60% recurrent pre-eclampsia. Active management can also reduce the incidence of severe recurrent pre-eclampsia, reduce the risk of delivering an SGA infant and prevent stillbirth/neonatal death in patients with recurrent pre-eclampsia. However, there were no controls who did not receive active management in this study. Our findings need to be further confirmed by a case-control or a randomized clinical trial study.

## Data Availability Statement

The raw data supporting the conclusions of this article will be made available by the authors, without undue reservation.

## Ethics Statement

The studies involving human participants were reviewed and approved by the Ethics Committee of First Hospital of Xi'an Jiaotong University, China (ref 2018-115). The patients/participants provided their written informed consent to participate in this study.

## Author Contributions

XD and XL: involved in patient recruitment reported in this work. MH and SZ: data analysis. MT: involved in manuscript draft. XL and QC: designed study and wrote the manuscript draft. All authors were involved in the drafting, editing, and approval of the manuscript for publication.

## Conflict of Interest

The authors declare that the research was conducted in the absence of any commercial or financial relationships that could be construed as a potential conflict of interest.

## Abbreviations

BMI: Body mass index
SGA: Small for gestational age
ISSHP: International Society for the Study of Hypertension in Pregnancy
ACOG: The American College of Obstetricians and Gynecologists.
